# Adherence to antiretroviral therapy in a clinical cohort of HIV-infected children in East Africa

**DOI:** 10.1371/journal.pone.0191848

**Published:** 2018-02-21

**Authors:** Rachel C. Vreeman, Samuel O. Ayaya, Beverly S. Musick, Constantin T. Yiannoutsos, Craig R. Cohen, Denis Nash, Deo Wabwire, Kara Wools-Kaloustian, Sarah E. Wiehe

**Affiliations:** 1 Indiana University School of Medicine, Indianapolis, IN, United States of America; 2 Regenstrief Institute, Inc, Indianapolis, IN, United States of America; 3 Academic Model Providing Access to Healthcare (AMPATH), Eldoret, Kenya; 4 School of Medicine, College of Health Sciences, Moi University, Eldoret, Kenya; 5 R.M. Fairbanks School of Public Health, Indianapolis, IN, United States of America; 6 University of California San Francisco, San Francisco, CA, United States of America; 7 City University of New York (CUNY) Graduate School of Public Health and Health Policy, New York, NY, United States of America; 8 Makerere University–Johns Hopkins University Research Collaboration, Kampala, Uganda; Aklilu Lemma Institute of Pathobiolgy, Addis Ababa University, ETHIOPIA

## Abstract

**Objective:**

To describe antiretroviral therapy (ART) adherence and associated factors for a large HIV-infected pediatric cohort followed by sites of the East Africa International Epidemiologic Databases to Evaluate AIDS (IeDEA) consortium.

**Methods:**

This study utilized prospectively collected clinical data from HIV-infected children less than 13 years of age who initiated ART within 4 clinical care programs (with 26 clinical sites) in Kenya, Uganda, and Tanzania and were followed for up to 6 years. Programs used one of 3 adherence measures, including 7-day quantitative recall, 7-day categorical recall, and clinician pill assessments. We fit a hierarchical, three-level, logistic-regression model to examine adherence, with observations nested within patient, and patients within the 26 sites providing pediatric HIV data to this analysis.

**Results:**

In East Africa, 3,304 children, 52.0% male, were enrolled in care and were subsequently observed for a median of 92 weeks (inter-quartile range [IQR] 50.3–145.0 weeks). Median age at ART initiation was 5.5 years ([IQR] 3.0–8.5 years). “Good” adherence, as reported by each clinic’s measures, was extremely high, remaining on average above 90% throughout all years of follow-up. Longer time on ART was associated with higher adherence (adjusted Odds Ratio–aOR–per log-transformed week on ART: 1.095, 95% Confidence Interval–CI–[1.052–1.150].) Patients enrolled in higher-volume programs exhibited higher rates of clinician-assessed adherence (aOR per log-500 patients: 1.174, 95% CI [1.108–1.245]). Significant site-level variability in reported adherence was observed (0.28), with even higher variability among patients (0.71). In a sub-analysis, being an orphan at the start of ART was strongly associated with lower ART adherence rates (aOR: 0.919, 95% CI [0.864–0.976]).

**Conclusions:**

Self-reported adherence remained high over a median of 1.8 years in HIV care, but varied according to patient-level and site-level factors. Consistent adherence monitoring with validated measures and attention to vulnerable groups is recommended.

## Introduction

The advent of antiretroviral therapy (ART) has transformed HIV infection from a rapidly terminal illness into a chronic disease. ART suppresses HIV replication, reduces HIV morbidity and mortality, and improves the lives of HIV-infected children [1–5] but successful long-term treatment depends on adherence to ART regimens. Patients who do not maintain adequate adherence to ART are at greater risk of viral resistance to the available antiretroviral drugs,[6–10] of immunologic decline resulting in opportunistic infections[11] and HIV disease progression,[12–17] and of transmitting resistant HIV at sexual debut.[18] Good ART adherence, typically defined as 90% or greater of doses taken as prescribed, decreases children’s HIV-related morbidity and mortality.[13, 19–21]

Over 90% of the world’s 3.4 million HIV-infected children live in the resource-limited settings of sub-Sahara Africa.[22, 23] While children’s access to ART in these settings is rapidly expanding, many patients still do not have access to second-line ART regimens and even fewer have access to third-line regimens.[24] Guidelines for pediatric HIV treatment recommend immediate ART initiation for HIV-infected infants, which should significantly reduce mortality among young children, but older children are expected to continue to have growing AIDS mortality.[25] Non-adherence and subsequent viral resistance could eliminate children’s limited treatment options.

Data from sub-Saharan Africa are still accumulating, but current estimates of pediatric ART adherence in resource-limited settings have ranged widely, with reports that 24.6% to 100% of their children have perfect adherence.[26–42] Despite this wide range, the majority of studies from resource-limited settings report good adherence among HIV-infected children; 76% of the studies included in a systematic review of pediatric ART adherence in low- and middle-income countries reported greater than 75% ART adherence, but few had been treating children for more than 3 years.[26] Most of these studies used caregiver reports of adherence, but self-reported adherence among older children or adolescents may yield reports of more non-adherence. In a study from Zimbabwe, 39% of adolescents reported suboptimal adherence, but significantly fewer reported suboptimal adherence if guardians were present in the room [37]. Other studies in sub-Saharan Africa have been able to prospectively measure adherence with more intensive and typically more valid methods, but these evaluations usually involve smaller numbers of children and collect adherence data prospectively for research. Such methods include home-based pill counts [35, 40], clinic-based pill counts [39], medication event monitoring systems (MEMS®) [36, 42, 43], plasma drug concentrations [38] and hair drug concentrations [41]. Few treatment programs in sub-Saharan Africa have been able to report on children’s adherence over the long-term or within large cohorts. In addition, these studies primarily focus on individual-level factors associated with adherence, without consideration of site- or facility-level contributions.

The objective of this study was to describe pediatric ART adherence for a large clinical cohort of HIV-infected children in East Africa. Data from the East Africa International Epidemiologic Databases to Evaluate AIDS (IeDEA) consortium were used to estimate ART adherence and to investigate both patient-level and site-level factors associated with ART non-adherence.

## Methods

### Study design

This study used prospectively collected, de-identified data from the computerized medical records of HIV-infected, pediatric patients treated in 26 sites belonging to 4 HIV care and treatment programs affiliated with the East Africa IeDEA consortium in Kenya, Uganda, and Tanzania until December 31, 2010. We sought to describe physician-assessed pediatric adherence, whether self-reported, proxy-reported, or assessed by the clinician via other methods, and associated patient or site-level factors. Each participating pediatric HIV clinic formally agreed to be included in the IeDEA collaboration, with local Institutional Review Board approvals.

### Study sites

Established in 2006, IeDEA is a global data consortium funded by the United States National Institutes of Health to develop 7 regional data centers to compile global data to address research questions about HIV/AIDS treatment and care that cannot be answered with individual cohorts (see www.iedea.org).[44] The East Africa IeDEA regional Consortium involves clinics providing HIV care and treatment in Kenya, Uganda, and Tanzania. Four of these clinical care programs provide care for children and adolescents (at 26 clinc sites) and are included in this analysis. These four programs are: 1) Academic Model Providing Access to Healthcare (AMPATH) clinical care system in western Kenya (AMPATH consists of 25 clinics and 40 satellite clinics; 17 of the main clinic sites provide pediatric care)[45], 2) Family AIDS Care and Educational Services (FACES) in Kisumu, Kenya (FACES included 4 clinic sites, Family Health Options Kenya–FHOK, Lumumba, Pandpieri, and Tuungane)[46]; 3) the Mother-to-Child Transmission-Plus (MTCT-Plus) program (with 3 clinic sites, the Nyanza Provincial General Hospital in Kisumu, Kenya, the Makerere University–Johns Hopkins University Research Collaboration (MUJHU) in Kampala, Uganda and the St. Francis Hospital–Nsambya in Kampala, Uganda)[47]; and 4) the Tanzania Ministry of Health Community Development, Gender, Elderly and Children (MoHCDGEC) (with 2 clinic sites, Morogoro Regional Hospital in Morogoro, Tanzania; and the Tumbi Special Hospital in Kibaha, Tanzania). For the purpose of this analysis, AMPATH, FACES, MTCT-plus, and MoHCDGEC are refered to as *programs*, where as the individuals clinics within reach program are referred to as *sites*, In addition, any satellite clinic data from the AMPATH program were incorporated into their main “parent” clinic, which provides pediatric medical supervision.

Over the course of 10 years of collaboration in IeDEA, the programs have standardized protocols for data collection related to pediatric HIV care. However, the East Africa IeDEA sites each followed their own, local clinical protocols and procedures during the time period of the analysis and the systems may have implemented the protocols differently or to varying degrees. HIV-infected children were typically seen at least every three months. During the time period of this study, children’s CD4 counts were supposed to be measured once or twice a year for immunologic monitoring. Routine viral load monitoring was not performed at any of the sites at the time of these analyses. All sites had access to first-line antiretroviral therapy (ART) for children.

### Ethical considerations

This analysis was approved as part of the projects of East African IeDEA by the Indiana University institutional research ethics board (IRB) as well as the local, and where appropriate, national regulatory bodies affiliated with the participating sites and regional data centers. As the analysis utilizes de-identified data collected within routine care, the local institutional research ethics boards or committees did not require written informed consent for patient-level data to be utilized in this analysis.

### Study population

Eligible patients included HIV-infected children who had at least one medical visit at any of the pediatric HIV care sites of the East Africa IeDEA consortium between January 2002 and January 2009; had initiated ART; and had at least one ART adherence measure recorded in the electronic medical record database prior to 13 years of age. In most of these sites, children above 13 receive care within the adult clinics. Thus, the study population was limited to those still considered “children” at the time of the adherence assessment across the various care programs. Child anthropometric data were categorized according to the WHO Growth Standards [48] and orphanhood was defined using the standard definition of having a mother dead, a father dead, or both [49].

### Data collection and adherence measures

A computerized clinical data collection system with a common set of variables for all sites was established for the participating IeDEA HIV care programs. Both site-level and patient-level data were extracted from the electronic medical record for this study and validated following established standard operating procedures within the East Africa IeDEA collaboration. The mechanisms for receiving and combining data from individual sites, including training on data collection; processing and cleaning; verifying data quality; and implementing methods for analyzing cohort data are all standardized.

The clinical sites recorded adherence data using one of 3 different adherence measures: a 7-day quantitative recall, a 7-day categorical recall of any missed pills, or a clinician pill assessment. Whether the recall items were asked of the child or the caregiver was not specified for the data within the electronic database. Clinical protocols at the sites using the recall methods generally direct the clinician to ask the child’s caregiver about adherence (proxy-report) unless the child is alone or the caregiver indicates that the child is primarily responsible for taking his/her own medicines, in which case the child should be asked directly (self-report). For the 7-day quantitative recall, adherence data were collected from responses to the question, “During the last 7 days, how many doses of his/her antiretroviral medicines did the patient take?” with potential response options of: “none,” “few,” “half,” “most,” and “all.” Good adherence was defined as reporting that “all” doses were taken. This adherence measure was used at the AMPATH and MTCT-plus sites. For the 7-day categorical recall of any missed pills, the caregiver or patient was asked “Has the patient missed any of his/her pills during the last 7 days?” and the potential responses were only “yes” or “no”. This measure was used by the FACES sites, and good adherence was equivalent to a “no” response. Adherence was measured through a clinician pill assessment for the Tanzanian MoHCDGEC sites, where clinicians asked to see the medication remaining when a child presented for a return visit, did a visual check of the medication, and then recorded whether the amount of remaining medicine indicated good adherence (defined in their protocol as pills remaining suggesting two or fewer missed doses over the past month) or poor adherence (pills remaining suggesting three or more missed doses over the past month.) The clinician pill assessment did not quantify how many pills are remaining, as would be usual for a standard “pill count.” These three different types of ART adherence measures were collapsed into a binary measure of “good” versus “poor” ART adherence per visit for all sites to create a summary outcome assessing the adherence across these sites.

### Statistical analyses

We used descriptive statistics to describe this cohort of East African children at enrolment into care and at ART initiation. Adherence (“good” vs. “poor”) was described for various time periods after ART initiation, by site and by a number of factors including demographics, patient volume at the site (expressed as the number of patients within a site having recorded visits at each time point) and treatment course (expressed as weeks from ART initiation). Program attrition was defined as not having a visit for any reason for more than 90 days since database closure. The database did not distinguish between the end of observation due to mortality or treatment disengagement. Measures of time from ART initiation were censored at the time of attrition, and estimates of the probability of remaining on follow-up were produced using the Kaplan-Meier method. Conversely, times from ART start were censored at database closure or reaching one’s 13^th^ birthday, and program attrition was considered the event of interest when estimates of program retention (defined as one-minus the probability of attrition) were calculated. The effect of adherence on program attrition was assessed via a Cox model with multiple adherence evaluations per patient considered as time-dependent covariates.

We fit a hierarchical, three-level logistic regression model, with observations nested within patients and patients within sites (sites were each of the 26 clinics belonging to the 4 East Africa IeDEA pediatric care programs participating in this study). We report the relative variability associated with the level “site” and the level “patient” to quantify the relative proportion of variability attributed to each factor. A few other patient-level and site-level factors were also used in the exploratory data analysis. With regard to patient-level factors we concentrated on age and gender as well as the duration of exposure to antiretroviral therapy. We also examined the effect of patient volume (number of patients per site at the same time point) as a proxy of program size, maturity but also burden on human and non-human resources. Statistical analyses were performed using R version 3.4.2 (2017-09-28) (the R Foundation for Statistical Computing).

## Results

### Characteristics of eediatric HIV care in East Africa

The East Africa IeDEA cohort included 3,304 HIV-infected children who had been started on ART at the care systems in Kenya, Uganda, and Tanzania (Table 1). Of the total cohort, the majority (79.1%) were followed at AMPATH sites in western Kenya. The median age at initiation of ART was 5.5 years (inter-quartile range [IQR] 3.0–8.5 years; Table 1). Just over half of the children (52.0%) were male. Data on orphan status were only collected for children followed at the AMPATH sites. Of the 1,962 children with orphan status known, 55.5% had at least one parent dead at initiation of ART. At their last visit, the children had been receiving ART for a median of 91.7 weeks (IQR 47.1–91.7 weeks). Program attrition (loss to follow-up or death) was 15.8% at one year post-ART initiation (95% CI: 14.5%-17.1%) and 24.3% (95% CI: 22.5%-26.0%) at two years. In a Cox analysis with good adherence considered as a time-dependent covariate (i.e., an analysis involving multiple observations per patient), the average (over all observations) impact of perfect adherence on the time until program attrition was to slightly reduce the hazard by almost 25% (hazard ratio 0.77, 95% CI 0.53–1.11). However, this reduction was not statistically significant (p-value 0.160).

**Table 1:** Characteristics of East African HIV-infected children on antiretroviral therapy (N = 3,165).

Population Characteristics	N (%)
**Age at ART Initiation**	
< = 1 year	171 (5.2%)
1–3.0 years	669 (20.2%)
3.0–5.0 years	666 (20.2%)
5.0–7.0 years	587 (17.8%)
7.0–9.0 years	507 (15.3%)
9.0–11.0 years	385 (11.7%)
11.0–13.0 years	290 (8.8%)
**Median Age (years) at ART Initiation**	5.5 (IQR 3.0–8.5 years)
**Gender**	
Male	1717 (52.0%)
Female	1587 (48.0%)
**Orphan Status at ART Initiation (AMPATH only)**	N = 1,962
At Least 1 Parent Dead	1089 (55.5%)
Both Parents Alive	873 (44.5%)
**Tuberculosis at ART Initiation**	
On Anti-TB Treatment	578 (18.3%)
Not on Anti-TB Treatment	2587 (81.7%)
**Median Weeks on ART (95% CI)**	91.7 (IQR 88.0–95.1)

### Pediatric ART adherence in East Africa

The measures of adherence used by AMPATH, FACES, MTCT-Plus and the Tanzania MoHCDGEC programs, along with patient characteristics within each program, are listed in Table 2. Duration of observation after ART initiation varied greatly within program, with MTCT-Plus patients being observed the longest (median duration of observation 174 weeks, inter-quartile range–IQR– 112.1–230.9), while FACES patients were observed for the shortest duration (median 28.4 weeks, IQR 11.4–56.0 weeks).

**Table 2:** Measures of adherence, patient characteristics and duration of follow-up by program in East Africa.

	HIV Care & Treatment Program			
	AMPATH	FACES	MTCT-Plus	TZ MoHCDGEC
**N**	2613	190	268	233
**Adherence Measure Used At Site**	7 day	7 day, any missed	7 day	1 month pill count
**Median Age at ART Initiation (IQR)**	5.6 (3.2–8.5)	5.2 (2.5–8.8)	2.5 (0.9–5.9)	7.1 (4.0–10.1)
**Median weeks on ART (95% CI)**	90.7 (48.0–126.1)	28.4 (11.4–56.0)	174 (112.1–230.9)	92 (70–110)
**Male children (%)**	1372 (52.5%)	98 (51.6%)	135 (50.4%)	112 (48.1%)

**Fig 1:**
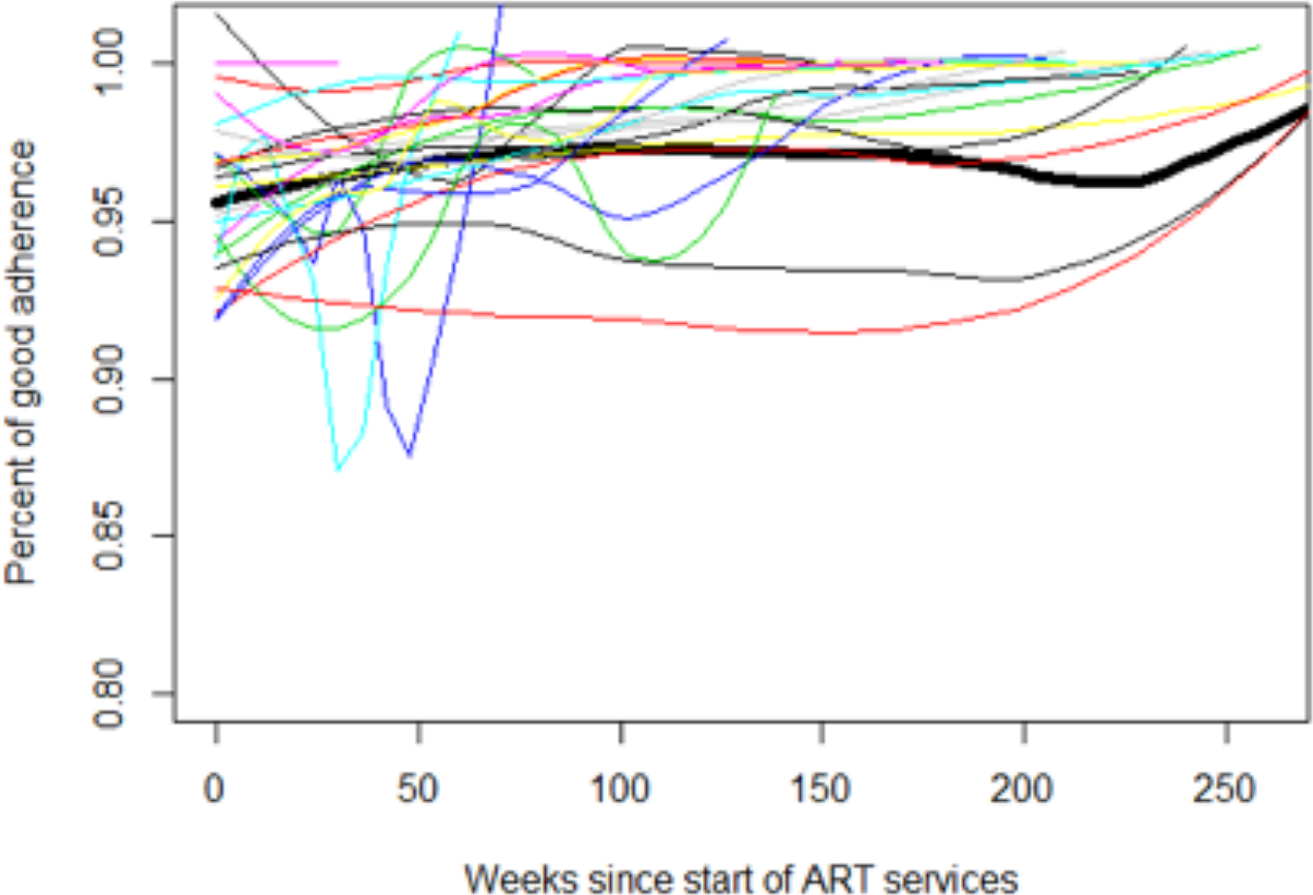
Time trend of mean adherence by site (colored loess curves) and overall trend (thick black line).

**Fig 2:**
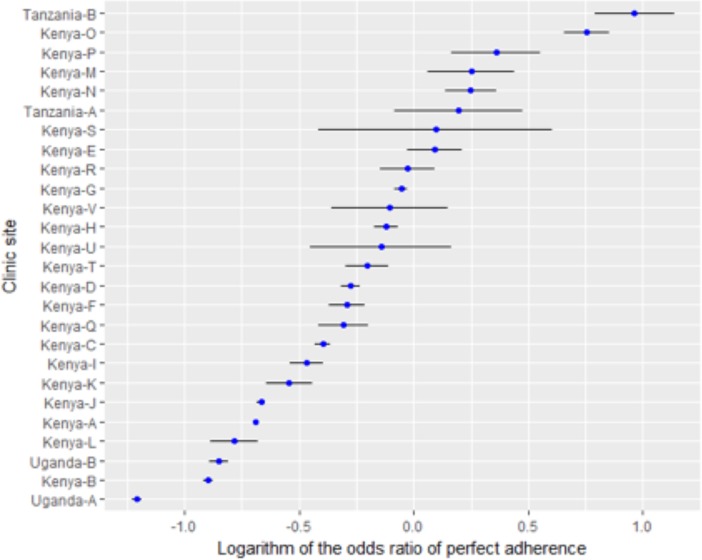
Log odds ratio of adherence and 95% confidence interval by site. Sites to the right of 0, are more likely than average to have good adherence, while sites to the left of 0 are less likely than average to have good adherence.

Overall, the rates of pediatric adherence among the East Africa sites remained stable over time, at an average above 90% (range 93.6%-98.4%) throughout all years of follow-up, with a slight upward trend (Fig 1, black line). The average rates of “good” adherence by six-month period, after ART initiation, by site and overall are described in Fig 2. The sites in Tanzania, which used the clinician pill assessment for adherence measurement, had the highest reported levels of adherence. The overall trend was dominated by AMPATH (the largest care program).

In the hierarchical model, with observations nested within patients and patients within sites, the variance in reported adherence associated with the site (0.28) was about a third of the variance associated with individual patients (0.71). (Table 3). Longer time on ART was associated with slightly higher adherence (aOR of the log-transformed week on ART 1.10, 95% Confidence Interval [1.005–1.015]; p-value <0.001). (Table 3) There was no difference in adherence depending on the gender of the child or the age at the start of ART. Reported adherence was higher within the programs that saw a higher volume of pediatric patients (Table 3).

**Table 3:** Multivariate hierarchical model of the odds of good adherence with observations nested within patients and patients within clinical sites.

Effect of …	OR	95% CI	p-value
Weeks on ART*	1.100	1.052–1.150	<0.001
Gender: Male	0.943	0.835–1.065	0.344
Age at ART Start (per year)	0.986	0.968–1.004	0.118
Volume of Patients (in 500-Patient Increments)*	00.852	0.803–0.903	<0.001

* Log-transformed

### Sub-analysis by orphan status

Orphan status was collected only at the start of therapy and only at AMPATH. Consequently, analyses assessing the impact of orphan status on ART adherence were limited to sites within this program. Results of the multi-level logistic-regression model applied in this analysis (with observations nested within AMPATH clinic sites) are shown in Table 4. Orphan status is associated with significantly lower odds of good adherence (aOR: 0.78, 95% Confidence Interval [0.64–0.95]). The impact of the remaining factors considered was consistent as described above for the complete data analysis.

**Table 4:** Sub-analysis assessing the effect of orphan status on ART adherence (AMPATH sites only). Multivariate hierarchical model with observations nested within patients and patients within AMPATH clinics.

Effect of …	OR	95% CI		p-Value
Weeks on ART*	1.190	1.100	1.287	<0.001
Gender: Male	1.120	0.941	1.334	0.200
Age at ART Start (per year)	0.995	0.967	1.024	0.718
Volume of Patients (in 500-Patient Increments)*	1.256	1.026	1.538	0.027
Orphaned at initiation of ART	0.919	0.864	0.976	0.012

* Log-transformed

## Discussion

The combined data from pediatric HIV care programs across East Africa suggest that the majority of HIV-infected children in these settings have good ART adherence on clinical assessments over a fairly long follow-up period (Fig 2). This level of adherence is similar to adherence reported over varying time periods within smaller cohorts of patients from East Africa [50–52] and elsewhere in sub-Saharan Africa.[29, 33] Our analyses of a larger cohort, with comparisons between sites and evaluation of adherence over time, indicates good ART adherence while suggesting some critical targets for measuring, supporting and improving adherence.

While ART adherence among East African children was high overall, certain groups may be prone to non-adherence. Better adherence among younger children suggests that adherence may become worse as children grow older, highlighting the potential challenges of maintaining good ART adherence during adolescence.[53] However, this study include predominately younger children, with only a weak suggestion of diminishing adherence with age. Following adherence closely as perinatally-infected pediatric cohorts age into adolescence is a key priority. These data did highlight that the sites with larger patient volumes had more reports of good pediatric adherence. This finding related to high-volume sites merits closer examination. It may be that these sites coping with large patient volumes do not actually have better adherence, but are, in fact, not detecting poor adherence. A site with over-burdened clinicians and support staff may not have the time or resources to thoroughly assess adherence with individual families. On the other hand, high-volume sites may be more experienced in detecting and managing families’ struggles with pediatric adherence, resulting in true improvements in adherence. The larger, referral centers also often provide care for a sicker population of children, creating a form of referral bias, and it is possible that they pay closer attention to adherence for these sicker children at risk of treatment failure. These data do not allow conclusions to be drawn on this issue.

These analysis provide a unique opportunity to assess the variability in adherence that could be attributed to site-level differences, in comparison with the individual patient-level factors typically evaluated. The variance in reported adherence associated with the site was almost one third as high as the variance associated the individual patients, even after all site-level factors were accounted for. This observation points that there are significant program-level factors not accounted for in these data, but also underscore the fact that, in addition to varying patient characteristics, program-level characteristics are critical in maintaining or improving adherence. The odds of good adherence increased *exponentially* with increasing volume of patients, for example. Addressing pediatric adherence not just within the context of a given child and caregiver, but as a pervasive clinical program issue appears to be a key strategy for maintaining our gains in the treatment of HIV-infected children over the long-term.

These analyses reveal some important issues related to adherence measurement. The sites that used pill counts reported much higher estimates of adherence, suggesting that the pill counts may not have accurately captured instances of non-adherence as higher adherence estimates are typically considered less accurate.[54] These findings may seem surprising since, in many reports, pill counts have been considered more accurate than recall measures [35, 39, 40, 55, 56]. However, the pill counts described across the East Africa clinical sites in this analysis were unlike the pill counts used in research settings. In this study, using data from routine clinical care, the actual number of pills dispensed at each visit and any pills remaining at the next visit were not recorded. Thus, even though the adherence measurement was called a “pill count”, there were at best only visual checks of the number of pills remaining, which in turn may have reduced the validity of these measures. Moreover, the sites did not use a specific method for weighing or estimating the volume of liquid medications, which can alter the accuracy of a “pill count” for all of the pediatric patients on liquid formulations. Pill counts were not directly compared with self-reports or proxy-reports in the sites taking part in this study, so no comparison can be made as to the pill count validity compared to other measures. Furthermore, the settings using recall measures did not employ any other comparative measures of adherence, such as pill counts or electronic dose monitoring. Although proxy-reports or self-reports are the most widely used method of adherence assessment, these reports also tend to overestimate adherence [35, 37, 38, 40, 42, 43, 54]. No attempt was made to compare the levels of adherence as measured by the three methods because no patient was evaluated with more than one method. Thus, the method and the site effects were confounded (i.e., any differences in the levels of adherence could not be reasonably disentangled from the effect that the sites had on adherence). Reliable, validated measures of ART adherence for children are urgently needed. The impact of orphanhood on adherence could only be assessed within the AMPATH program in Kenya, as this was the only program collecting data on the orphan status of children. The sub-analyses of this large sub-population of children suggested that orphanhood is another factor that may be associated wth reduced adherence levels. Given the critical role of the caregiver in administering medicines to children and in supervising medication-taking as children age, in combination with the potential economic and social upheaval that come with the death of a child’s parent or parents, this is not a surprising finding. The potential impact of orphanhood on children’s adherence points to the need to find strategies to support this vulnerable population, as well as to ensure measurement of orphan status for children across sub-Saharan Africa. A final limitation concerns trend toward increasing rates of good adherence over time. It is possible that, since adherence measures are taken only from patients returning to clinic, overall adherence may be overestimated as we begin to sample from the smaller group of patients that is retained in care over time. In the Cox analysis we performed to assess the possible relationship of imperfect adherence with the hazard of program attrition, however, we did not observe a strong association between lower adherence and higher hazard of loss to program or mortality.

In addition to limitations with respect to measuring adherence, this study has several further limitations that require consideration. We did not evaluate the relationship of clinical outcomes such as World Health Organization (WHO) or Centers of Disease Control (CDC) staging, CD4 counts or percentages to the self-report or proxy-reports of adherence. The clinical outcomes for children cared for within these sites were difficult to assess with accuracy from the available data. Viral load measurements were not available at these sites during the time of this study. While CD4 counts and clinical staging should have been collected or performed for most of the enrolled children, there were a paucity of data for these outcomes from many of the sites. Because of the gaps in the CD4 results in the clinical data, we chose to report adherence alone across the broad range of children, rather than to restrict the analyses to only those with complete CD4 data which would have severely limited our study population (and potentially have biased our results). The limitation of lacking CD4 comparisons is somewhat allayed by findings in other studies where reported adherence was not consistently associated with CD4 counts.[26, 57] The clinical staging data, while contained in these clinical data, are less useful for comparisons with adherence because patients always remain at the highest level of clinical staging that they have reached regardless of response to therapy. Good adherence, even if it results in an improved clinical picture, would thus fail to move a patient to a lower or less severe clinical stage.

Our data were limited to those variables available within the common dataset across the East Africa IeDEA sites. Viral loads were not available, nor were some other variables of potential importance, such as caregivers’ HIV status, orphan status for all children, and over time, or orphan care setting. Our study may also have underestimated the extent of ART non-adherence because adherence was based on in-clinic assessments and thus potentially compounded by clinic non-adherence or social desirability biases. While patients may have some extra medication supplies and should report non-adherence from missed visits during their subsequent visits, clinic-based ART assessments likely underestimate ART non-adherence. In addition, the nature of the observational cohort precludes us from making causal inferences.

Despite these limitations, this study has critical advantages that add to the current literature on pediatric ART adherence. The study involves a large cohort from an entire world region, across multiple programs, caring for children and following them for a number of years. The children in the analysis span a large spectrum of ages, and their general characteristics suggest that they are representative of a typical population of HIV-infected children in East Africa if not throughout sub-Saharan Africa. The representativeness of our population is also buoyed by the fact that we have captured data from both large and small care systems, while maintaining this wide regional representation. Consequently, these data allow for both care systems and policy makers to consider the challenges to pediatric ART adherence as we attempt to maintain the survival of an entire generation, from infancy and into adulthood living with HIV.

## Conclusions

Effective disease management for children with HIV depends on sustaining high rates of adherence. Although ART adherence appears to be very good among children in East Africa, HIV care systems in resource-limited settings must continue to measure, sustain, and improve ART adherence. Reliable, valid, affordable measurement strategies that can be employed for routine adherence measurement are urgently needed.
